# Exploring the Role of Visual Guidance in Motor Imagery-Based Brain-Computer Interface: An EEG Microstate-Specific Functional Connectivity Study

**DOI:** 10.3390/bioengineering10030281

**Published:** 2023-02-21

**Authors:** Tianjun Wang, Yun-Hsuan Chen, Mohamad Sawan

**Affiliations:** 1 Center of Excellence in Biomedical Research on Advanced Integrated-on-Chips Neurotechnologies (CenBRAIN Neurotech), School of Engineering, Westlake University, Hangzhou 310030, China; 2 School of Biological Science and Medical Engineering, Southeast University, Nanjing 210096, China; 3 Institute of Advanced Technology, Westlake Institute for Advanced Study, Hangzhou 310030, China

**Keywords:** EEG, motor imagery, guided motor imagery, microstate, microstate-specific functional connectivity

## Abstract

Motor imagery-based brain–computer interfaces (BCI) have been widely recognized as beneficial tools for rehabilitation applications. Moreover, visually guided motor imagery was introduced to improve the rehabilitation impact. However, the reported results to support these techniques remain unsatisfactory. Electroencephalography (EEG) signals can be represented by a sequence of a limited number of topographies (microstates). To explore the dynamic brain activation patterns, we conducted EEG microstate and microstate-specific functional connectivity analyses on EEG data under motor imagery (MI), motor execution (ME), and guided MI (GMI) conditions. By comparing sixteen microstate parameters, the brain activation patterns induced by GMI show more similarities to ME than MI from a microstate perspective. The mean duration and duration of microstate four are proposed as biomarkers to evaluate motor condition. A support vector machine (SVM) classifier trained with microstate parameters achieved average accuracies of 80.27% and 66.30% for ME versus MI and GMI classification, respectively. Further, functional connectivity patterns showed a strong relationship with microstates. Key node analysis shows clear switching of key node distribution between brain areas among different microstates. The neural mechanism of the switching pattern is discussed. While microstate analysis indicates similar brain dynamics between GMI and ME, graph theory-based microstate-specific functional connectivity analysis implies that visual guidance may reduce the functional integration of the brain network during MI. Thus, we proposed that combined MI and GMI for BCI can improve neurorehabilitation effects. The present findings provide insights for understanding the neural mechanism of microstates, the role of visual guidance in MI tasks, and the experimental basis for developing new BCI-aided rehabilitation systems.

## 1. Introduction

One of the numerous applications of the brain–computer interface (BCI) is assisting in activating neuroplastic mechanisms through various feedbacks in rehabilitation, in particular for stroke patients [1]. BCI rehabilitation systems are considered better than traditional rehabilitation because they are closed-loop and customized [2]. In post-stroke rehabilitation fields, action simulations, e.g., motor imagery (MI), motor observation (MO), and visually guided motor imagery (GMI), are widely studied since they have proven their ability to be used for neurological rehabilitation [3,4]. The mechanism of MI, MO, and GMI tasks’ application in stroke rehabilitation is that they can motivate neural reorganization processes, and the rehabilitation effect can be improved by increasing the co-activation of the motor network [1].

Among BCI modalities, electroencephalography (EEG) is frequently used because of its high temporal resolution, noninvasiveness, and portability. EEG signals have shown an excellent ability to classify different motor conditions [5] and different motions during MI tasks [6,7]. In the former studies of EEG, many investigations have been focused on static features, which may lose important temporal information. While dynamic analysis methods are believed to reflect brain activity patterns better and include information on the spatiotemporal dimensions [8]. EEG microstate analysis has been increasingly utilized for investigating the spatiotemporal properties of brain dynamics with multichannel EEG. The numbers of electrodes vary from 19 to 204 according to a previous review [9]. EEG microstates were first found by Lehmann et al. and were considered “basic building blocks of brain information processing” [10]. According to the microstate theory, the broad-band of EEG signals could be modeled as a time sequence of a finite number of discrete microstates that remain stable for 60–120 ms [9]. The EEG microstates were found to be mostly determined by alpha wave (8.5–12 Hz) [11]. In terms of describing brain connectivity, dynamic functional connectivity (dFC) analysis is a popular method. The sliding-window method was used in traditional dFC analysis [12]. However, the optimized window size is difficult to determine, and a fixed window size hardly reflects the cognitive processing stage [12]. To overcome the limitations, microstate-specific functional connectivity (MSFC) analysis was proposed. It calculates the functional connectivity (FC) pattern of each microstate. Since microstates were considered to be associated with different cognitive processing stages in many studies [9,10], MSFC provides more exhaustive and reliable information on brain dynamics. It has been previously used for studying brain signals in stroke patients [8], during cognitive tasks [13], during resting-state [14], during mental workload [15], and influenced by continuous theta-burst stimulation [16].

In neurorehabilitation fields, GMI was proposed because it was hypothesized that MI could be affected by external cues to reach a better rehabilitation effect [17]. The visually guided MI in this study can also be seen as a combination of MO and MI. Previous studies have provided evidence based on oxyhemoglobin (HbO) responses [18], imagery vividness scores and eye movements [17], and event-related desynchronization (ERD) values [3]. However, to our knowledge, no research published has provided microstate or functional connectivity evidence in support of the hypothesis.

Most existing microstate studies focused only on one or two motor conditions among the MI, ME, and GMI. Some former studies [19,20,21] conducted microstate analysis for MI-EEG, proving the ability of microstate parameters as classification features. Fu et al. [22] focused on EEG microstate patterns related to executed and imagined grip. However, the brain microstate patterns activated by MI, GMI, and ME and their comparisons remain unstudied. Besides, to our knowledge, no previous research has studied the relationship between the FC patterns and any specific microstates in motor-related tasks. To summarize, we expect to understand motor-related tasks, especially GMI, from a new perspective by exploring how the microstate and MSFC patterns change with different motor conditions.

In this study, the EEG data were collected from 30 healthy participants under 18 tasks. Each task is a combination of one of the six motions (i.e., right-hand finger tapping, left-hand finger tapping, holding a pen, opening a pen, crossing fingers, and moving an arm) under one of the three motor conditions (i.e., MI, GMI, ME). After that, we conducted microstate analysis and investigated the stability and consistency of microstates when analyzing different numbers of tasks. Next, we analyzed four types of microstate parameters: mean duration, duration, coverage, and occurrence. Then, we studied the accuracy of the ME versus MI and ME versus GMI classification tasks using those microstate parameters. Last, we conducted the MSFC analysis based on graph theory. These results prove that the microstate parameters are useful for comparing brain dynamics during various motor conditions. Furthermore, the classification accuracy between a given motor condition and ME with a microstate parameter-based SVM classifier can be used for creating a quantitative index for individually evaluating the potential rehabilitation effect. This index can also be used as feedback in a closed-loop BCI system. Moreover, brain activation patterns of those three motor conditions are analyzed and new evidence supporting rehabilitation therapy to combine MI and GMI is proposed. In short, the main contribution of the present study is providing new microstate-based references for designing and constructing BCI-aided rehabilitation systems.

In the remaining parts of this paper, we introduce in Section 2 the proposed methods of EEG signal acquisition and processing. The results are shown in Section 3 and discussed and analyzed in Section 4. The conclusions are the subject of Section 5.

## 2. Materials and Methods

### 2.1. Experiments and Data Acquisition

A total of thirty healthy subjects were recruited (15 males and 15 females, 29 are right-handed; aged 24.26 ± 3.46 years). All subjects declared no history of stroke or other brain diseases. The experiment was approved by Westlake University Ethics Committee (approval ID: 20191023swan001). EEG signals of MI, ME, and GMI were recorded during six motions: right-hand finger tapping, left-hand finger tapping, holding a pen, opening a pen, crossing fingers, and moving an arm. Each task included five trials under three motor conditions (ME, MI, GMI). In MI tasks, subjects were instructed to imagine themselves doing the motion without any muscle activities with audio stimuli. In GMI tasks, the screen shows a picture of a specific motion, and subjects were instructed to conduct MI tasks with visual guidance.

EEG data were collected with 32 Ag/AgCl electrodes, a ground electrode, and a reference electrode, arranged in accordance with the 10–20 international standard. The EEG recording system consisted of the Brain Products actiCHamp Plus (EEG signal amplifier) and actiCAP slim (active EEG electrodes). More details of the recording system of the EEG signals and the experimental protocol can be found in our former work [5]. The dataset of this manuscript and our former work is the same.

### 2.2. Preprocessing

**Figure 1:**
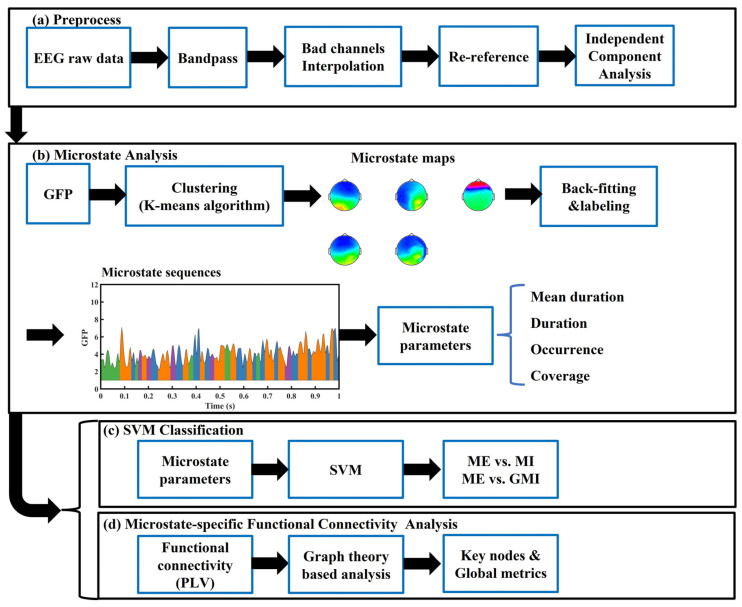
Flow-process diagram of the analytical process in this study: (**a**) EEG signal preprocess procedures, (**b**) Microstate analysis procedures, (**c**) SVM classification procedures, (**d**) Microstate-specific functional connectivity analysis procedures. EEG: electroencephalography; GFP: global field power; GMI: guided motor imagery; ME: motor execution; MI: motor imagery; PLV: phase lock value; SVM: support vector machine.

Figure 1 shows a flow-process diagram of all analytical processes in this study. Preprocessing includes the following procedures: First, raw data were bandpass filtered (1–45 Hz) with finite impulse response (FIR) filters to remove signals that are not in the interested frequency range. Signals with a larger frequency range than what is needed in the current study were retained because they might be used in our future research. Second, we down-sampled the EEG signals to 250 Hz to reduce the calculation time. Next, bad channels and segments, whose signals were evidently polluted by noise or with abnormal power spectrum, were investigated visually and removed. Subjects with less than six bad channels were selected for further analysis because a former study had shown that the error of interpolation increases with the increment of the number of bad channels [23]. This method gave us nine subjects. This sample size is similar with former studies of microstate analysis of MI-EEG [20,21]. All retained subjects are right-handers. Next, we interpolated the bad channels’ signal with spherical interpolation before re-referencing all EEG data to the common average. After that, independent component analysis (ICA) was used for decomposing the EEG data, and artifactual components, including eye and muscle components, were discarded via visual inspection. Lastly, all trials were epoched between −1 and 3 s with baseline correction, resulting in 810 epochs. EEGLAB toolbox (version 2022.0) [24] and MATLAB (R2022a) were applied to conduct all preprocessing procedures above.

### 2.3. EEG Microstate Analysis

Microstate analysis was conducted in the same procedures as some former microstate studies [8,21,22]: global field power (GFP) calculation, microstates clustering, back-fitting, labeling, and microstate parameters calculation. Microstate and MSFC analyses were conducted with a MATLAB toolbox: +microstate ([25]; plus-microstate.github.io).

First, an 8–15 Hz bandpass filter (BPF) was used to get the EEG signal around the alpha band (8–13 Hz). Alpha and beta (13–30 Hz) bands are most commonly used in MI-BCI [26]. In a previous study of microstate analysis of MI and ME [22], the alpha band shows the highest correlation coefficient value with the clustered microstates. Besides, this specific frequency band was chosen by a former study involving the microstate analysis of MI-EEG [21] and achieved good results. After this, EEG data were re-referenced to the common average.

GFP of EEG signals was calculated according to Equation (1) [25]. GFP is used to quantify the amount of activity [27]. The value of GFP is defined as the value of the standard deviation of the EEG signal. Next, the topographies with the highest signal-to-noise ratio (SNR) were extracted based on local maximal values of the GFP [28].

(1) $$\mathrm{GFP}=\frac{\sqrt{\left(\sum_{i}^{N}{\left(V_{i}\left(t\right)-V_{\mathrm{mean}\;}\left(t\right)\right)}^{2}\right)}}{N_{e}-1}$$

where $V_{i}\left(t\right)$ is the potential value at time t of the i th channel, $V_{\mathrm{mean}\;}\left(t\right)$ is the mean potential value at time t of all channels, and $N_{e}$ is the channel number [29].

Next, we applied the K-means clustering method to find the optimized microstate maps with the original topography maps. This method is widely adopted in the former microstate-related studies [8,22]. After that, we ran the clustering analysis for 2–7 states to optimize the number of microstate maps. In all conditions considered in this work, the optimum number is five. Then, the microstate maps were back-fitted to the preprocessed EEG signal and a temporal microstate sequence was given by labeling original signals based on maximal similarity to the microstate maps. A detailed description of the above methods and algorithms can be found in the toolbox manual [25].

### 2.4. Microstate Parameters Calculation

Four microstate parameters were calculated and compared in this work. (a) mean duration of microstates: mean value of the time all microstate maps lasted [28]; (b) coverage: the time ratio of signal in each microstate map [28]; (c) occurrence: the frequency of each microstate map appears [30]; (d) duration: the mean value of the time each microstate map lasted [30]. Since we have five microstates, duration, coverage, and occurrence were calculated for all five microstates separately, and mean duration is the mean value of the five microstates. In total, sixteen parameters were computed for each task.

### 2.5. Microstate-Specific Functional Connectivity Analysis

MSFC was calculated by the “+microstate” MATLAB toolbox. We adopted the alpha band (8–13 Hz) phase lock value (PLV) to measure FC between every two channels. The PLV value between the two channels was calculated based on Equation (2) [31]:

(2) $$PLV_{i,j}^{m}=\left|\frac{1}{N}\overset{N}{\underset{n=1}{\sum }}e^{i\left(\varphi_{jn}-\varphi_{in}\right)}\right|$$

where N is the number of time points of a specific microstate m, and $\varphi_{jn}$, $\varphi_{in}$ are the phase angles of point n from channels i and j, which was obtained by the Hilbert transform. The microstate-specific PLV were only calculated if the total duration of the microstate is longer than 500 ms, because the PLV value was considered unreliable if the data length was shorter than 5 cycles of the central frequency [32]. In our study, 10 Hz is the central frequency of the relevant frequency bands, so 500 ms is the shortest acceptable microstate total duration per epoch.

Next, a weighted brain network was constructed for each microstate under every task to investigate the functional integration and segregation among different brain regions [33]. The set of nodes corresponds to all EEG channels, and the edge values are the mean PLV value between each channel pair.

After that, key nodes were calculated based on nodal betweenness centrality ($N_{bc}$), and $N_{bc}$ was calculated based on Equation (3) [34]:

(3) $$N_{bc}\left(i\right)=\underset{s\neq i\neq t}{\sum }\frac{\rho_{st}\left(i\right)}{\rho_{st}}$$

where $N_{bc}\left(i\right)$ is node i ’s nodal betweenness centrality value, $\rho_{st}\left(i\right)\;$ is the number of the shortest paths that pass the node i from node s to node t, $\rho_{st}$ is the total number of the shortest paths from node s to node t. The $N_{bc}$ of a given node characterizes its effect on information flow within the network. The nodes with high $N_{bc}$ ($N_{bc}\geq mean+SD$) were defined as key nodes because they are considered as the centers of a network and are important in the expression of information flow [35]. Besides, we calculated three global metrics: global efficiency ($E_{g}$), clustering coefficient ($C_{p}$), and characteristic path length ($L_{p}$). $E_{g}\;$ describes the efficiency of a network in transferring information between nodes. A brain network with a higher $E_{g}$ means a larger information-transferring speed between brain regions. It was calculated based on Equation (4) [15]:

(4) $$E_{g}=\frac{1}{N_{n}}\underset{i\in Ns}{\sum }E_{i}=\frac{1}{N_{n}}\underset{i\in Ns}{\sum }\frac{\sum_{j\in N_{s},j\neq i}d_{ij}^{-1}}{N_{n}-1}$$

where $N_{s}$ is the set of all nodes, $N_{n}$ is the number of nodes in the network, $E_{i}$ is the local efficiency of the node i, and $d_{ij}$ is the shortest path length from node i to j. Nodal $C_{p}$ describes the likelihood its neighborhoods are connected to each other. Mean $C_{p}$ is the average value of all nodal $C_{p}$. A brain network with a higher mean $C_{p}$ contains higher local information processing. Mean $C_{p}$ was calculated as Equation (5) [15]:

(5) $$C_{p}=\frac{1}{N_{n}}\underset{i\in Ns}{\sum }C_{i}=\frac{1}{N_{n}}\underset{i\in Ns}{\sum }\frac{2T_{i}}{d_{i}\left(d_{i}-1\right)}$$

where $C_{i}$ is the individual nodal $C_{p}$ of the nodes, $T_{i}\;$ is the count of triangles through node i, and $d_{i}$ is node i’s degree which is defined as Equation (6):

(6) $$d_{i}=\underset{i\in Ns}{\sum }e_{ij}$$

where $e_{ij}$ is the weight of the edge between node i and j.$\;L_{p}\;$ was defined as the global average shortest path length. A brain network with a shorter $\;L_{p}$ has higher information transformation efficiency. $L_{p}$ was calculated as shown in Equation (7) [15]:

(7) $$L_{p}=\frac{1}{N_{n}}\underset{i\in Ns}{\sum }L_{i}=\frac{1}{N_{n}}\underset{i\in Ns}{\sum }\frac{\sum_{j\in N_{s\;},j\neq i}d_{ij}}{N_{n}-1}$$

where $\;L_{i}$ is the nodal shortest path length of node i, which is the mean shortest path length from node i to the other nodes within the network.

All graph metrics mentioned above were calculated with the GRETNA toolbox (v2.0.0) [36].

### 2.6. Support Vector Machine Classifier

The support vector machine (SVM)’s classification principle is finding a hyper-plane with maximal distance between classes within the training set in a high-dimensional space [37]. It has shown its applicability in classifying small training sets and nonlinear relationships [38]. In this study, we trained an SVM as the classifier between ME versus MI and GMI. Leave-one-out cross-validation was applied to get the accuracies.

### 2.7. Statistical and Visualization Tools

In this study, all significance level is set as *p* < 0.05. Two-way repeated-measures ANOVA (RANOVA, motion × motor condition) was applied in both microstate and MSFC analyses. Missing values of original data were replaced with average value of other data before statistical analyses. Before the RANOVA analysis, Mauchly’s test and Shapiro–Wilk test were applied to evaluate the sphericity and normality of input data, respectively. If the assumption of normality was not tenable, pairwise non-parametric permutation tests with 100,000 replications were directly applied. If the assumption of sphericity was not tenable, the severity of the sphericity problem could be measured by a statistic: ε, ranging from 0 to 1. The data were processed by Huynh–Feldt correction if ε ≥ 0.75. If ε < 0.75, data were corrected with Greenhouse–Geisser correction. To further explore the changes of measurements under each motion, one-way RANOVA (Factor: motor condition) was utilized. All multiple comparisons in this study were corrected with the Bonferroni method.

IBM SPSS Statistics 26.0 was used to conduct all statistical analyses mentioned above. Box diagrams were plotted with OriginPro 2022.

## 3. Results

### 3.1. Microstate Maps

**Figure 2:**
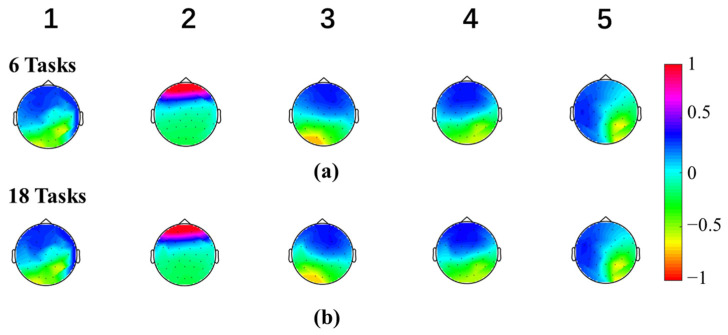
Five Microstate maps of two sets of tasks: (**a**) The microstates clustered in six tasks (right and left-hand finger tapping under MI, ME, and GMI conditions), (**b**) The microstates clustered in all eighteen tasks (right and left-hand finger tapping, holding a pen, opening a pen, crossing fingers, and moving an arm under MI, ME and GMI conditions). The value of the microstate topographies was normalized to the range between −1.0 and 1.0.

Microstate maps were calculated separately among six tasks (right and left-hand finger tapping under MI, ME, and GMI conditions) and all eighteen tasks (right and left-hand finger tapping, holding a pen, opening a pen, crossing fingers, and moving an arm under MI, ME and GMI conditions). Figure 2 shows the topographies of the microstate maps. It can be observed that the maps with the same microstate number derived from “6 tasks” and “18 tasks” are similar. The mean Pearson correlation coefficient value of all map pairs was 99.77% ± 0.20%, showing high consistency between the two sets of microstate maps.

### 3.2. Microstate Parameters Analysis

**Figure 3:**
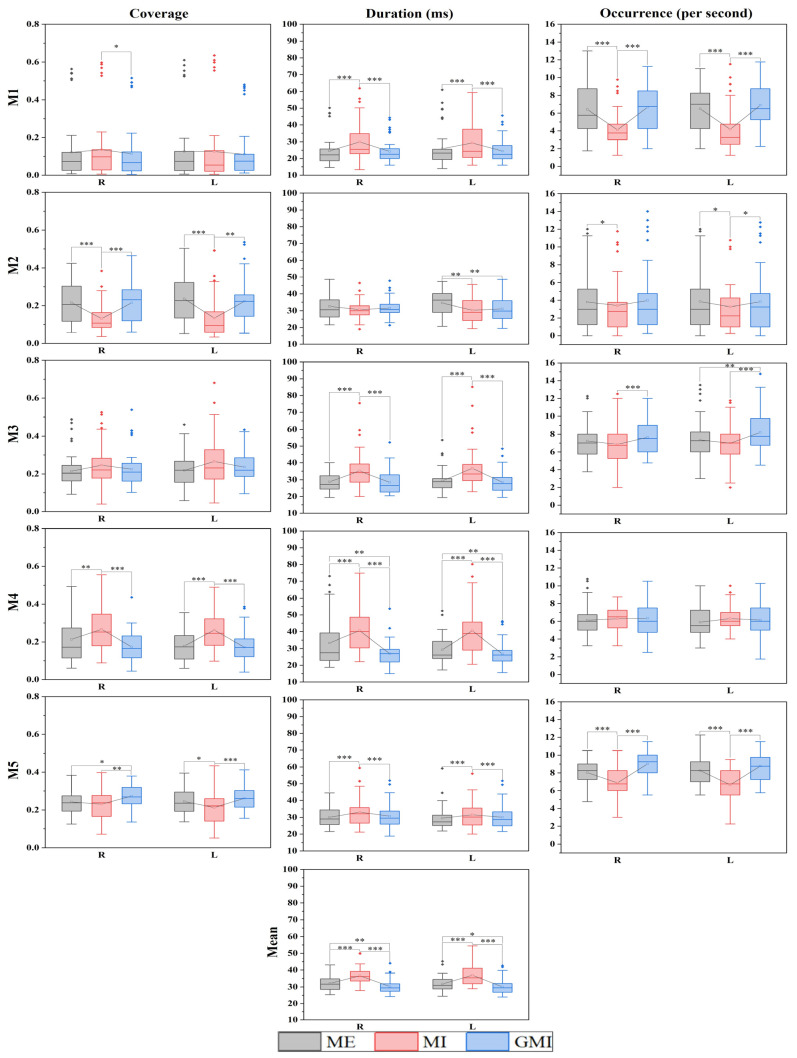
Analysis between tasks of microstate parameters of finger-tapping motions. The 25–75th percentiles are represented by the box. Mean value, median value and outliers are represented by the square, the central line, and the diamonds, respectively. R: right-finger tapping; L: left-finger tapping; Mn (*n* = 1, 2, 3, 4, 5): the *n*th microstate. * *p* < 0.05; ** *p* < 0.01; *** *p* < 0.001.

Mean duration, duration, coverage, and occurrence of the microstates were calculated. Duration, coverage, and occurrence were calculated for all five microstates individually, and mean duration is the mean value of the five microstates, resulting in a total of sixteen parameters. All parameters of finger-tapping motions are shown in Figure 3. The Mn stands for the *n*th microstate.

In Figure 3 the analysis between tasks of the microstate parameters of finger tapping motions is presented. In total, 24 pairs of parameters show a significant difference between MI and GMI. They are coverage of M1, M2, M4, M5, duration of M1, M3, M4, M5, occurrence of M2, M3, M5, and mean duration during right-hand finger tapping. In addition, the coverage of M2, M4, M5, duration of M1, M3, M4, M5, occurrence of M1, M2, M3, M5, and mean duration during left-hand finger tapping. Furthermore, 22 pairs of parameters show a significant difference between MI and ME. They are coverage of M2, M4, duration of M1, M3, M4, M5, occurrence of M1, M2, M5, and mean duration during right-hand finger tapping, as well as the coverage of M2, M4, M5, duration of M1, M2, M3, M4, M5, occurrence of M1, M2, M5, and mean duration during left-hand finger tapping. It is noted that only seven pairs of parameters show a significant difference between GMI and ME. This is shown in the coverage of M5, duration of M4, and mean duration during right-hand finger tapping, as well as duration of M2, M3, M4, and mean duration during left-hand finger tapping. All microstate parameters with a significant difference between all three pairs of motor condition are shown in Table 1, Table 2 and Table 3, respectively.

### 3.3. Classification between Motor Conditions

In Section 3.2, the result shows that GMI has a smaller number of significant different parameters with ME than MI. However, a more intuitive index is desired to evaluate the difference between a given motor condition and ME. Since the basic principle of SVM is to maximally separate the distributions of the input groups’ features in parameter space and determine an optimal hyperplane [39], the classification accuracy could reflect the degree of dissimilarity between two given tasks.

We adopted an SVM to test microstate parameters’ ability to discriminate motor conditions. We used the sixteen parameters discussed in the Section 3.2 as feature vectors to train the SVM. An accuracy of 80.27% was achieved for the classification between ME and MI tasks across all six motions. Note that we trained the modal with all data across motions, rather than training with data of separate motions and calculating the average accuracy. This confirms that the results are the reflection of only motor conditions and that this method is applicable to multiple motions. We also tested the classification accuracy between ME and GMI across all motions. An accuracy of 66.67% was achieved, indicating higher difficulty in distinguishing ME and GMI based on microstate features. To further investigate the relationship between classification performance and motion types, we tested the classification accuracy under each motion separately, and the results are shown in Table 4. According to Table 4 the average single motion classification accuracy of ME versus MI and ME versus GMI is 80.27% ± 6.50% and 66.30% ± 5.56%, respectively. The classification accuracy difference between the two conditions is 13.97% ± 9.25%.

### 3.4. Microstate-Specific Functional Connectivity

The results in Section 3.2 and Section 3.3 indicate that GMI could induce more similar brain activation patterns to ME than MI from the microstate analysis. Here, we further analyze MSFC across all 18 tasks. We calculated each microstate segment’s alpha band network (8–13 Hz). We adopted multivariate pattern analysis by using the *networks_mvpa* function in +microstate toolbox [25] to test the relationship between FC patterns and specific microstate classes. We obtained a *p*-value of 0.005, suggesting a strong association between microstate class and FC pattern.

**Figure 4:**
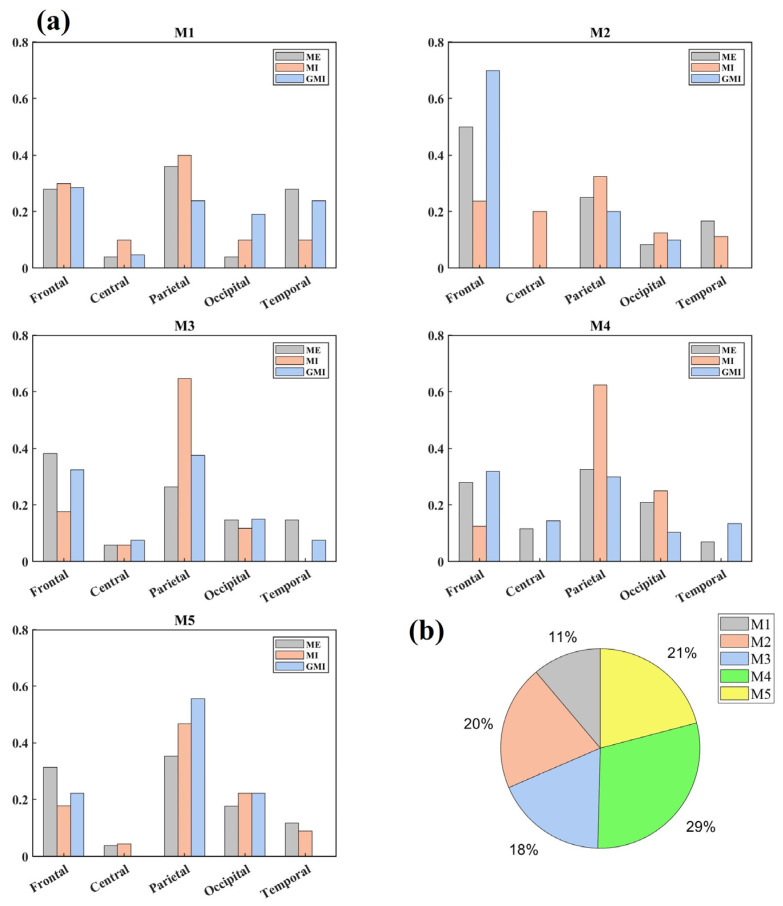
(**a**) Key nodes distributions in brain areas during each microstate; (**b**) Key nodes distributions in microstates. All distribution percentages are rounded to the nearest whole number. Mn (*n* = 1, 2, 3, 4, 5): the *n*th microstate.

Next, the key nodes were calculated. Figure 4a shows the area distributions of key nodes in each microstate. It was found that during ME tasks, the key nodes were mostly distributed in the parietal area (36.00%), frontal area (50.00%), frontal area (38.24%), parietal area (32.56%), and parietal area (35.29%) for microstates M1 to M5, respectively. During GMI tasks, the key nodes were mostly distributed in the frontal area (28.57%), frontal area (70.00%), parietal area (64.71%), frontal area (31.96%), and parietal area (55.56%) for microstates M1–M5, respectively. During MI tasks, key nodes were mostly located at the parietal area (40.00%, 32.50%, 37.50%, 62.50%, and 46.67%) for all microstates. Figure 4b shows the microstate distributions of key nodes for all tasks and brain regions. It can be concluded that half of the key nodes were distributed in M4 (29%) and M5 (21%).

**Figure 5:**
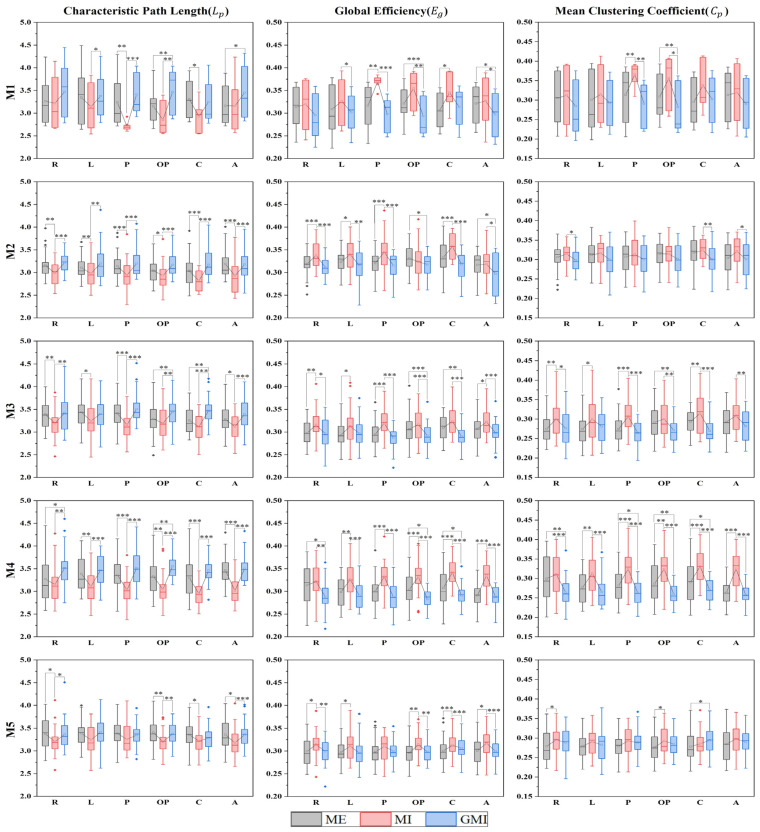
Global metrics of MSFC brain network under all tasks. The 25–75th percentiles are represented by the box. Mean value, median value and outliers are represented by the square, the central line, and the diamonds, respectively. R: right-finger tapping; L: left-finger tapping; P: holding a pen; OP: opening a pen; C: crossing fingers; A: moving an arm; Mn (*n* = 1, 2, 3, 4, 5): the *n*th microstate. * *p* < 0.05; ** *p* < 0.01; *** *p* < 0.001.

Three global metrics based on graph theory: $E_{g}$, $C_{p}$, and $L_{p}\;$ were calculated and analyzed. Two-way RANOVA showed that the three metrics in all microstates were significantly influenced by motor condition, and only four parameters ($E_{g}$ at M2, $C_{p}$ at M3, M4, and $L_{p}$ at M2) were significantly influenced by motions. To further explore the detailed influence of motor condition on global metrics, one-way RANOVA (Factor: motor condition) was conducted for each motion. Figure 5 shows the results under all 18 tasks.

For $L_{p}$, pairwise comparisons show that for all significant different pairs, $L_{p}\;$ during GMI is longer than ME, and $L_{p}\;$ during MI is lower than both ME and GMI. For $E_{g}$, pairwise comparisons show that for all significant different pairs, $E_{g}\;$ during GMI is lower than ME, $E_{g}\;$ during MI is higher than both ME and GMI. While for $C_{p}$, pairwise comparisons show that for all significantly different pairs, $C_{p}\;$ during MI is higher than both ME and GMI, and $C_{p}\;$ during GMI is lower than ME except for crossing fingers at M5.

## 4. Discussion

To study the brain activation patterns and evaluate the rehabilitation potential under MI and GMI tasks, microstate analysis and MSFC analysis were conducted on EEG data of multiple motions under ME, MI, and GMI conditions. First, we gave five microstate maps, showing high consistency between six and eighteen tasks. This suggests that the microstate maps are stable within our dataset and irrelevant to the dataset size and motion types. Though in most resting-state microstate studies, four types of microstate maps were given [9], the optimal cluster number of microstate maps in task states varies in different studies [22].

Next, from Figure 3, we gave microstate-based evidence that GMI could induce more similar brain activation patterns with ME than MI. Previous studies have shown that visual guidance of MI could enhance HbO responses [18], improve ERD values [3], and increase imagery vividness [17]. In our study, we provided new evidence from a microstate-based brain dynamic perspective showing the similarity of brain activation patterns between GMI and ME. Moreover, the duration of M4 and the mean duration of all microstates are significantly changed in all pairwise comparisons. Thus, the two parameters are most sensitive to the change of motor conditions, making them potential biomarkers in evaluating motor conditions.

Based on the difference in microstate parameters between motor conditions, we further tested the classification ability of the parameters between ME and MI or GMI. It should be noted that those accuracies were achieved without feature selection; we used all four types of microstate parameters directly as the feature vector. In EEG classification tasks, feature selection is a commonly used method to choose the best subset of features to reduce computation time and increase classification accuracy [40]. Feature selection methods, e.g., the relief algorithm, effect-size-based feature selection, and minimally redundant maximally relevant algorithm, have been used in EEG classification tasks and proved their ability to increase accuracy [41]. We did not apply feature selection because our goal in classifying motor conditions is not to reach high accuracy but to give an intuitive index to evaluate the given EEG signal’s similarity with the EEG pattern under the ME condition. Thus, selecting a limited subset of given features with the highest classification ability is not desired. All microstate features were retained. However, even without feature selection and using only microstate parameters, accuracy (80.27% across all six motions) higher than our former study [5] was achieved. In that study, an accuracy of 78.57% was achieved with 26 statistical, wavelet-based, and power parameters using the same dataset. Therefore, microstate parameters are efficient and stable features in classifying ME and MI tasks.

In this study, the lower the classification accuracy, the more similar the brain dynamics are to ME. As shown in Table 4, mean accuracy is 80.27% in MI versus ME classification but only 66.30% in GMI versus ME under each motion, showing higher similarity between GMI and ME with a quantitative index. This index could be part of the basis for creating a quantitative index for individually evaluating the potential rehabilitation effect. Moreover, the classification accuracy is decreased by 13.97% ± 9.25% because of the involvement of visual guidance. However, we can clearly see that the decrement of right-hand finger tapping (2.44%) and holding a pen (2.98%) is much smaller than other motions (left-hand finger tapping: 22.96%; opening a pen: 13.34%; crossing fingers: 16.88%; moving an arm: 24.24%). Besides, the classification accuracies for ME versus GMI of right-hand motions (right-hand finger tapping: 70.54%; holding a pen: 73.33%) are higher than other motions (left-hand finger tapping: 57.59%; opening a pen: 62.22%; crossing fingers: 67.08%; moving an arm: 66.67%). Since the majority of our subjects are right-handers, they are likely to be most familiar with dominant hand motions than nondominant hand, two hand, and arm motions. The evidence together proves that more complex and unfamiliar motions may be better assisted by visual guidance during MI tasks and are more useful in neurorehabilitation.

Finally, we conducted the MSFC analysis and constructed functional connectivity brain networks. Results of the key nodes analysis show clear switching of key node distribution between brain areas among different microstates. The switching and distribution of key nodes among microstates might be undetected in static FC analysis, which only gives one set of key nodes calculated from the overall functional connectivity. Since the key nodes with high $N_{bc}$ are considered to be critical in the information flow within a network, they are deemed the central part of the network [35]. These results indicate that, in different microstate periods, the brain may process different cognitive functions, and the central areas of brain networks switch accordingly. In our study, the switching of the key nodes between the parietal area and the frontal area at various microstates shows a high frontal–parietal integration. Similarly, Kincses et al. [42] reported that the fronto-parieto-cerebellar network was identified as a task-related component during sequential tapping tasks. Moreover, meta-analysis [43] identified a predominantly premotor-parietal network for MI and the cortical sensorimotor and premotor network for ME, which explains why predominant key nodes distributions are consistently in parietal areas for MI, but switching between Frontal and Parietal areas for ME. What’s more, GMI’s predominant key nodes distributions in frontal in M1, M2, M4 can be explained by the mirror neurons (MNs). MNs were found in area F5 of the ventral premotor cortex located in the frontal lobe, and the inferior parietal lobule [44]. These areas were found to be more closely associated with MO than MI [43]. Since during the GMI tasks, subjects were instructed to conduct MI while observing the visual-guidance, GMI is likely to cause the cognitive process of MO.

Former studies of resting-state microstates have found an association between EEG microstates and resting state networks [45]. Four microstates in resting-state conditions are proven to be associated with the auditory network, saliency network (SN), visual network, and attention network [46]. We speculate that microstates during motor-related tasks are associated with brain networks of different cognitive functions as well. In our study, the midline frontal-occipital topography pattern of M2 is very similar to the microstate C observed in resting-state condition, which was proved to be correlated with SN [9,46]. SN was believed to play an important role in responding to internal or external stimuli concerning homeostasis and the coordination of neural resources [47]. Thus, the significant higher coverage and occurrence of M2 during GMI than MI may be attributed to the SN’s higher activation in processing visual stimuli. The evidence listed above indicates that the increment of duration and occurrence of M2 and the distribution proportion of key nodes in frontal areas can at least in part be explained by SN’s activation in processing visual stimuli and the MNs’ activation in MO brought by visual guidance.

Besides, the M1 in our study with left-frontal right-posterior topography is similar to the microstate B observed in resting-state condition, which was proven to be correlated with the visual network [9,46]. Accordingly, from Figure 4a, GMI has the highest key node distribution in the Occipital area during M1 (ME: 4.00%, MI: 10.00%, GMI: 19.05%). M1 is also the only microstate during which GMI has a higher key node distribution proportion than the other two motor conditions. Thus, the evidence indicates that the cognitive progress during M1 is related to visual stimuli.

While the topographies of M4 and M5 are not typically found in resting state studies, they contribute to half of the key nodes’ distribution (M4: 29% and M5: 21%), indicating their importance in motor-related tasks. Thus, we speculate they are task-specific microstates. Both of them have a high proportion of key nodes distribution in frontal and parietal areas. This distribution of key nodes fits the former findings that the interaction between posterior parietal regions and frontal motor regions is activated in both MI and ME tasks [48]. Notably, the topography of M5 has a clear lateralized pattern between the left and right hemispheres. Therefore, M5 may contribute in the processing of one-hand motions, e.g., right and left-finger tapping, which have lateralized brain activation patterns [49].

The results of global metrics of MSFC brain network show an overall higher $E_{g}$, higher $C_{p}$, and shorter $L_{p}$ for MI compared with both ME and GMI. All three global metrics consistently indicate a higher functional integration and information flow intensity of the brain network during MI tasks. This may be attributed to the higher mental workload during MI task. A similar change in global metrics was reported in [15] when the mental workload increases. What’s more, though visual guidance of MI can induce similar brain activation patterns from a microstate perspective, the brain network functional integration is reduced, hindering neurorehabilitation [1,50]. Thus, we speculate that compared with single task of MI or GMI, the combination of these two tasks may lead to better rehabilitation results. As proven by He et al. [51], after GMI training, the EEG signals during MI tasks show enhancement in various features. However, more experiments need to be conducted on patients to confirm this hypothesis.

In this study, by conducting microstate and MSFC analyses on EEG signals during ME, GMI, and MI tasks, we explored the role of visual guidance in MI tasks for multiple motions. In Table 5, we compared this work with other published works that study motor-related tasks with a microstate approach.

In Table 6, we compared this work with other published works that study GMI with different approaches. To our knowledge, we achieved the first systematic study that conducts microstate and MSFC analysis on MI, GMI, and ME tasks under multiple motions. Microstate-based biomarkers are first proposed to evaluate motor conditions. Moreover, we have the highest motion number in relevant studies. The motions in our study contain one-hand motion, two-hand motion, and arm motion, making our results more comprehensive. However, this study still has several limitations, and more work needs to be done in the future. As mentioned above, all subjects formally analyzed in this study are right-handers. Future experiments for left-handers need to be performed to examine whether the results of this work are universal. What’s more, only healthy subjects were recruited in our study. To further study the rehabilitation ability and neural mechanism for GMI and MI, we need to conduct the same experiment in stroke patients. Additionally, we only focused on the alpha band of EEG. Though lower than the alpha band, beta band EEG signals also showed a high correlation coefficient value with the clustered microstates in a previous study [22]. The MSFC patterns in a larger frequency band can also be explored.

## 5. Conclusions

To sum up, the present study conducts EEG microstate and MSFC analyses in MI, GMI, and ME conditions for the first time. We first give evidence that visual guidance of MI could help induce more similar brain activation patterns with ME in a microstate-based brain dynamics perspective. We compared the microstate parameters between motor conditions and proposed duration of M4 and mean duration as biomarkers for motor condition evaluation. Furthermore, an average accuracy of 80.27% for ME versus MI and 66.30% for ME versus GMI classification tasks for each motion were achieved by using an SVM. Dominant hand motions were found to benefit less from visual guidance than nondominant hand, two hand, and arm motions. These results provide new references for accurately evaluating brain dynamic similarity between ME and given motor condition, which could assist in evaluating the expected rehabilitation effect. Finally, through MSFC analysis, we proved that the FC patterns are paired with specific microstates. We found the dynamic key nodes distribution among microstates which might be undetected in static FC analysis, and further discussed the neurophysiological mechanism behind microstates. In the end, we proved that visual guidance may reduce the information flow intensity and functional integration in MI tasks. Combining with the former results, we proposed new evidence supporting a BCI rehabilitation system to combine MI and GMI tasks. Therefore, this work provides new empirical evidence on the role of visual guidance in MI tasks from a microstate perspective, advances the understanding of microstates in motor-related tasks, provides a new reference for motion selection, and has further implications for research on BCI and neurorehabilitation.

## Figures and Tables

**Table 1:** Microstate parameters with significant difference for comparison between MI and GMI.

Motion	Microstate Parameters			
	Coverage	Duration	Occurrence	Mean Duration
Left-hand finger tapping	M2, M4, M5	M1, M3, M4, M5	M1, M2, M3, M5	√
Right-hand finger tapping	M1, M2, M4, M5	M1, M3, M4, M5	M2, M3, M5	√

**Table 2:** Microstate parameters with significant difference for comparison between MI and ME.

Motion	Microstate Parameters			
	Coverage	Duration	Occurrence	Mean Duration
Left-hand finger tapping	M2, M4, M5	M1, M2, M3, M4, M5	M1, M2, M5	√
Right-hand finger tapping	M2, M4	M1, M3, M4, M5	M1, M3, M5	√

**Table 3:** Microstate parameters with significant difference for comparison between ME and GMI.

Motion	Microstate Parameters			
	Coverage	Duration	Occurrence	Mean Duration
Left-hand finger tapping		M2, M3, M4		√
Right-hand finger tapping	M5	M4		√

**Table 4:** Classification accuracy (in %) of ME versus MI and ME versus GMI under six motions.

	Right-Finger Tap	Left-Finger Tap	Hold a Pen	Open a Pen	Cross Fingers	Arm Movement	Mean ± STD
ME vs. MI	72.98	80.91	77.31	75.56	83.96	90.91	80.27 ± 6.50
ME vs. GMI	70.54	57.95	73.33	62.22	67.08	66.67	66.30 ± 5.56
Accuracy difference	2.44	22.96	3.98	13.34	16.88	24.24	13.97 ± 9.25

**Table 5:** A comparison of studies that investigate motor-related tasks with microstate approach.

Authors	Main Results	Classification Study	FC Analysis	Motor Conditions			Specific Biomarkers	Motion Number
				MI	ME	GMI		
Liu et al., 2017 [21]	Mean accuracy of 89.17% was achieved for two motion classification using microstate-based features.	Used SVM to classify motions.	×	√	×	×	×	2
Li et al., 2021 [20]	Mean accuracy of 93.93% was achieved for two motion classification using microstate and Teager energy operator features.	Used SVM to classify motions.	×	√	×	×	×	2
Fu et al., 2018 [22]	Discussed the change of microstate parameters between ME and MI of grip tasks; proved alpha wave has the highest correlation with microstates.	×	×	√	√	×	×	3
Kim et al., 2020 [19]	Topography of M5 in their study can be used as a biomarker for errors in MI-BCI.	×	×	√	×	×	Topography of M5.	2
This work	Duration of M4 and mean duration can be biomarkers to evaluate motor condition; SVM classifier can be used to quantitatively evaluate motor condition difference; GMI could induce similar brain activation pattern with ME, but may reduce the functional integration of the brain network.	Used SVM to classify motor conditions.	√	√	√	√	Duration of M4 and mean duration.	6

FC: functional connectivity; BCI: brain-computer interface.

**Table 6:** A comparison of studies that investigate GMI with different approaches.

Authors	Main Results	Classification Study	FC Analysis	Motor Condition				Specific Biomarkers	Motion Number
				MI	ME	GMI	MO		
Romano-Smith et al., 2019 [52]	After GMI training, task performance was significantly increased compared to MI and MO interventions.	×	×	√	×	√	√	×	1
He et al., 2019 [51]	After GMI training, EEG signals during MI tasks show enhancement in various features, Common Spatial Pattern is most significant, indicating improved spatial resolution.	×	×	√	×	√	×	Characteristics of Common Spatial Pattern	1
Almulla et al., 2022 [18]	Analyzed with functional near-infrared spectroscopy signals, GMI activated greater HbO responses compared MI or MO alone.	×	×	√	×	√	√	HbO response	2
Rungsirisilp et al., 2022 [3]	GMI can induce higher ERD values in sensorimotor area and achieve better classification performance than MI.	Used SVM to classify motions.	×	√	×	√	×	ERD/ERS values of channel C3 or C4	2
This work	GMI induces similar brain activation pattern with ME than MI; Dominant hand motions are less benefit from visual guidance than nondominant hand, two hand, and arm motions; the brain network is less integrated during GMI than MI.	Used SVM to classify motor conditions.	√	√	√	√	×	Duration of M4 and mean duration.	6

HbO: oxyhemoglobin; ERS: event-related synchronization; ERD: event-related desynchronization.

## Data Availability

The data presented in this study are available from the corresponding authors, Y.-H.C. and M.S., upon request. The data are not publicly available because they contain information that could compromise the privacy of research participants.
